# Gastrisches versus supragastrisches „belching“, Singultus, Aerophagie und Differenzialdiagnosen: Eine interdisziplinäre Übersicht für den HNO-Arzt

**DOI:** 10.1007/s00106-024-01495-y

**Published:** 2024-06-27

**Authors:** Daniel Runggaldier, Roman Adam, Chiara Ermanni, Ursula Colotto-Vith, Miriam E. F. van Beek, Carsten Posovszky, Franziska Righini Grunder, Daniel Pohl, Jörg E. Bohlender

**Affiliations:** 1 Klinik für Otorhinolaryngologie, Head and Neck Surgery, Abt. Phoniatrie und Klinische Logopädie, Universitätsspital Zürich, Frauenklinikstrasse 24, 8091 Zürich, Schweiz; 2 Universität Zürich, Zürich, Schweiz; 3 Klinik für Gastroenterologie und Hepatologie, Universitätsspital Zürich, Zürich, Schweiz; 4 Gastroenterologie, Hepatologie und Ernährung, Universitäts-Kinderspital Zürich, Zürich, Schweiz; 5 Gastroenterologie, Hepatologie und Ernährung, Kinderspital Zentralschweiz, Luzern, Schweiz; 6 Klinik für Otorhinolaryngologie, Head and Neck Surgery, Universitätsspital Zürich, Frauenklinikstrasse 24, 8091, Zürich, Schweiz

**Keywords:** Regurgitation, „Rülpsen“, Hiccups, Reflux, 24h Impedanz pH Metrie, Regurgitation, Burping, Hiccups, Reflux, 24-hour pH-Impedance Testing

## Abstract

Das als „belching“ bezeichnete Aufstoßen von Luft aus dem Ösophagus oder Magen in den Mund- und Rachenbereich gilt als physiologischer Prozess. Es kann jedoch in einem vermehrten Maße auftreten und im Sinne einer „belching disorder“ für die betroffenen Patienten mit einem erheblichen Leidensdruck verbunden sein. Die Diagnosestellung gestaltet sich zum Teil schwierig, insbesondere in Hinblick auf die Abgrenzung zu verwandten Krankheitsbildern wie der Aerophagie, dem Ruminationssyndrom oder dem Singultus. Neben der Diagnosestellung stellt auch die Therapie dieser Störungsbilder für den Hals-Nasen-Ohren-Arzt eine Herausforderung dar. Ziel dieser Arbeit ist es, eine interdisziplinäre Übersicht zu diesen Krankheitsbildern zu schaffen und klinisch-praktische Gesichtspunkte zur Diagnosestellung und Therapie aufzuarbeiten.

## Abstract (en)

Belching is the act of expelling air from the stomach or esophagus into the pharynx. Although the process is regarded as physiological, excessive belching might be associated with a significant burden for affected patients in the sense of a belching disorder. Diagnosis of a belching disorder is often challenging, and its differentiation from other conditions such as rumination syndrome, singultus, or aerophagia can be difficult. Treatment of these disorders also represents a challenge for otorhinolaryngologists. Hence, the aim of this review is to provide an interdisciplinary overview of these clinical syndromes and provide practical guidance for their diagnosis and treatment.

## Definition, Pathogenese und Ätiologie des „belching“

Das im englischen Sprachgebrauch als „belching“ (Synonyme: Ruktus, Eruktationen, umgangssprachlich: Rülpsen) bezeichnete Aufstoßen von Luft aus dem Ösophagus oder Magen in den Mund- und Rachenbereich gilt als physiologischer Prozess, der in der Regel bei fast allen Menschen beobachtet werden kann [26, 30]. Gelegentlich wird dies jedoch von den betroffenen Menschen als störend wahrgenommen und geht mit einer erheblichen Einschränkung der Lebensqualität einher. Gemäß ROME-IV-Kriterien ist ein pathologisches „belching“ durch ein störendes Aufstoßen von Luft an mindestens 3 Tagen pro Woche gekennzeichnet. Zudem muss für die Diagnosestellung nach den ROME-IV-Kriterien die Symptomatik über mindestens 3 Monate bestanden haben (mit einem Beginn mindestens 6 Monate vor Diagnosestellung) [14, 15]. Ist der Ösophagus der Ursprungsort für das Aufstoßen, so besteht definitionsgemäß ein „supragastric belching“, im Fall des Magens liegt ein „gastric belching“ vor [14, 15]. Gemäß der Literatur führt vor allem das „supragastric belching“ zu einem Leidensdruck, während das „gastric belching“ oftmals für die Betroffenen weniger oder nicht belastend ist [30].

Wesentliche Einblicke in die Mechanismen dieser beiden Arten des „belching“ konnten in den letzten Jahren vor allem durch die Entwicklung und Fortschritte bei der hochauflösenden Manometrie und der Impedanz-pH-Metrie gewonnen werden [30, 65]: Beim klassischen „gastric belching“ wird beispielsweise durch den Schluckvorgang selbst oder durch die Aufnahme von kohlensäurehaltigen Getränken Gas im gastroduodenalen Bereich angesammelt. Im Rahmen von vagalen Reflexbögen kann es intermittierend zu transienten Relaxationen des unteren Ösophagussphinkters (TLESR) mit Entweichen dieser Gase in den Ösophagus kommen. Dort kann wiederum über weitere Reflexbögen eine Relaxation des oberen Ösophagussphinkters getriggert werden, wodurch die Gase weiter in den Rachen- bzw. Mundraum austreten können [23, 30, 67].

Demgegenüber werden in Bezug auf das „supragastric belching“ zwei unterschiedliche Pathomechanismen postuliert. Beim „Air-Suction-Prinzip“ wird anhand von manometrischen Befunden eine kurzzeitige Bewegung des Diaphragmas nach aboral beobachtet, welche zu einem negativen intrathorakalen Druck führen kann. Bei gleichzeitig getriggerter Relaxation des oberen Ösophagussphinkters kann folglich Luft anterograd aus dem Pharynxbereich in den Ösophagus entlang des negativen Druckgradienten gezogen werden. Aufgrund des verschlossenen unteren Ösophagussphinkters wird diese Luft wenige Augenblicke später wieder aus dem Ösophagus in den Pharynx in retrograde Richtung herausgepresst. Zu erwähnen ist, dass dieser Prozess sehr schnell abläuft und daher nicht mit einem normalen Schluckvorgang und der damit verbundenen Peristaltik in Verbindung steht [28, 30]. Der andere Mechanismus, der im Rahmen des „supragastric belching“ bei einigen Patienten beschrieben bzw. beobachtet worden ist, ist das sog. „Air-Injection-Prinzip“. Dabei wird manometrisch eine simultane Druckerhöhung am ehesten durch eine pharyngeale Kontraktion auf Höhe des Zungengrunds im Pharynx beobachtet, welche als treibende Kraft für den Lufteintritt in den Ösophagus gewertet werden kann. Auch hier wird kurze Zeit später (ebenfalls bei verschlossenem unterem Ösophagussphinkter) die Luft aus dem Ösophagus zurück in den Pharynx gepresst [8, 30].

Der Beginn eines „supragastric belching“ ist häufig von oberen gastrointestinalen Beschwerden begleitet wie beispielsweise Phasen eines ausgeprägten Völlegefühls [46, 62]. Es wird angenommen, dass die betroffenen Patienten versuchen, dieser gastrointestinalen Symptomatik durch gewisse Bewegungsmuster des Diaphragmas entgegenzuwirken. Dabei können sich bei längerem Verlauf auch Automatismen im Sinne eines „belching“ entwickeln, wodurch der eigentlich willkürlich gesteuerte Prozess nicht mehr als solcher erkannt wird [30]. Für die Annahme einer solchen funktionellen Problematik spricht auch, dass Schlaf oder eine Ablenkung der Patienten oftmals mit deutlich weniger supragastrischen „Belching-Ereignissen“ verbunden ist – eine Tatsache, die auch durch manometrische Untersuchungen objektiviert werden konnte [26, 30].

„Supragastric belching“ oder das Ruminationssyndrom kann im klinischen Alltag als eine gastroösophageale Refluxerkrankung (GERD) fehlinterpretiert werden – mit der Konsequenz einer fälschlicherweise initiierten frustranen Protonenpumpeninhibitor(PPI)-Therapie [73]. Dennoch legt die Literatur einen engen Zusammenhang des „supragastric belching“ mit einer GERD nahe. In einigen Studien konnte bei fast der Hälfte aller GERD-Patienten ein vermehrtes „supragastric belching“ beobachtet werden [30, 47]. In einer kürzlich veröffentlichten Arbeit konnte zudem mittels Impedanz-pH-Metrie gezeigt werden, dass knapp ein Drittel der Patienten mit einem „supragastric belching“ eine erhöhte Säurebelastung des Ösophagus im Sinne einer Refluxerkrankung aufweist. Auffällig an diesen Daten war, dass oftmals ein „Supragastric- Belching-Ereignis“ unmittelbar einem Refluxereignis vorangegangen ist, während ein umgekehrter Zusammenhang signifikant seltener beobachtet werden konnte [27]. Ähnliche Indizien haben sich in weiteren Studien ergeben [22, 34], weshalb eine kausale Triggerung von Refluxepisoden durch „Supragastric-Belching-Episoden“ diskutiert wird [31]. Insgesamt ist daher die Identifizierung und das Management der „Belching-Problematik“ bei Refluxpatienten in der klinischen Praxis vor allem bei fehlendem Ansprechen auf eine PPI-Therapie sinnvoll und könnte auch mit einem Benefit für die Betroffenen in Hinblick auf die Refluxbeschwerden verbunden sein [27, 47]. Weitere Studien sind jedoch erforderlich, um diese Zusammenhänge – insbesondere auch in Bezug auf einen möglichen laryngopharyngealen Reflux – besser zu verstehen.

Dem „belching“ ist die Entität der „inability to belch“ oder retrograden krikopharyngealen Dysfunktion (R-CPD) gegenübergestellt, welche durch eine Unfähigkeit zum Luftaufstoßen, abdominales Völlegefühl nach dem Essen, gurgelnde retrosternale Geräusche, exzessive Flatulenzen, Beeinträchtigung des sozialen Lebens und Schwierigkeiten beim Erbrechen definiert ist [2]. Die Therapie besteht dabei in einer Botulinumtoxin-Injektion in den M. cricopharyngeus und kann bei vielen Betroffenen zu einer deutlichen Besserung der Beschwerden führen [2, 25]. Eine Falldarstellung mit Kurzübersicht hierzu wurde kürzlich publiziert, sodass wir in diesem Artikel bewusst nicht weiter auf dieses Krankheitsbild eingehen [59].

### „Belching“ in der Pädiatrie

Mit der Einführung der Impedanz-pH-Metrie (pH-MII) haben pädiatrische Gastroenterologen neue Erkenntnisse zur Bedeutung des nichtsauren Refluxes und des „belching“ für die Symptomatik bei Kindern gewonnen. Nichtsaure Refluxepisoden treten bei Säuglingen und Kindern mit Refluxbeschwerden mit 40–89 % der Fälle sehr häufig auf und können mit vorangehender Aerophagie mit „belching“ oder „supragastric belching“ assoziiert sein [69]. Die Prävalenz der Aerophagie bei Kindern liegt bei 3–7 % [54]. Im Gegensatz dazu gibt es kaum Daten in Bezug auf die Prävalenz des „belching“ in der Pädiatrie. Bei symptomatischen Kindern, bei welchen die Symptomatik mittels pH-MII abgeklärt wurde, konnte ein „supragastric belching“ in 2,7 % der Fälle nachgewiesen werden – im Vergleich zu dem approximativen Wert von 3,4 % bei den Erwachsenen. Nur eine größere retrospektive Impedanz-pH-Metrie-Studie bei 287 Kindern unterscheidet zwischen „gastric“ oder „supragastric belching“ [42]. Das „gastric belching“ findet sich in dieser Studie vor allem bei Kindern mit erhöhter Säureexposition. Dagegen wird ein „supragastric belching“ nur sehr selten in dieser Kohorte nachgewiesen (1 %) und ist unabhängig von Refluxereignissen [42]. „Supragastric belching“ ist im Kindesalter häufiger gemeinsam mit einer Aerophagie beschrieben, im Gegensatz zu den Erwachsenen, wo diese Entität häufiger im Zusammenhang mit einer ineffizienten ösophagealen Motilität (IEM) oder einer GERD identifiziert wurde [42].

### Diagnostik

Ein wesentlicher Teil der Befunderhebung kann schon während der Anamneseerhebung durch gezielte Beobachtung des Patienten erfolgen: Charakteristischerweise zeigt sich ein „supragastric belching“ nicht beim sprechenden Patienten, sondern in den Phasen der Konsultation, in denen der Patient zuhört. Beim aktiven Sprechen oder bei einer gezielten Ablenkung kommt es nämlich oft zu einer Suppression dieser „Belching-Episoden“ [30]. Ebenfalls typisch für das „supragastric belching“ ist das Auftreten von aufeinanderfolgenden und repetitiven Episoden, während sich ein „gastric belching“ meist durch einzelne und isolierte Ereignisse auszeichnet [30]. Damit vereinbar haben Studien gezeigt, dass auch das Einbringen von größeren Mengen Luft in den Magen in der Regel nur einzelne, isolierte (gastrische) „Belching-Ereignisse“ zur Folge hat, wo jeweils auch größere Mengen Luft aufgestoßen werden können [6, 9].

Der Goldstandard zur Diagnosesicherung sind die hochauflösende Ösophagusmanometrie (HRM) sowie die 24-h-Impedanz-pH-Metrie, wobei Letztere eine genaue Erfassung und Analyse der „Belching-Episoden“ ermöglicht. Mittels HRM lassen sich die Druckverhältnisse während der „Belching-Episoden“ darstellen. Durch die 24-h-Impedanz-pH-Metrie können hingegen gasförmige und flüssige Volumenbewegungen im Ösophagus differenziert werden. Dabei wird die Impedanz an mehreren Stellen des Ösophagus gemessen und spiegelt dadurch den elektrischen Widerstand wider. Luft bewirkt eine Erhöhung, Flüssigkeit eine Erniedrigung der Impedanz. Zudem kann eine Aussage getroffen werden, ob sich Luft bzw. Flüssigkeit anterograd Richtung Magen oder retrograd vom Magen Richtung Pharynx bewegt [6, 9].

**Abb. 1:**
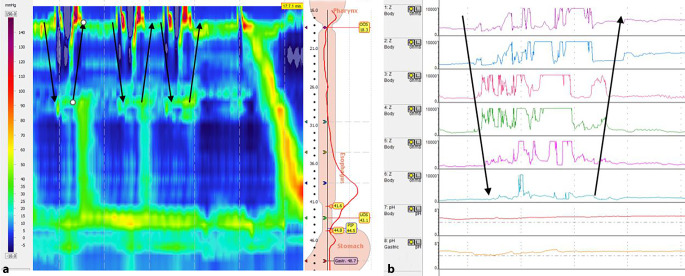
„Supragastric belching“. **a** Hochauflösende Manometrie mit „clouse plot“. Relaxation des oberen Ösophagussphinkters durch Hineinsaugen von Luft in den Ösophagus, wobei es nicht zu einer Relaxation des unteren Ösophagussphinkters kommt. Ausbreitung der Luft in Richtung der *Pfeile*. **b** 24-h-Impedanz-pH-Metrie mit anterograd verlaufendem Anstieg der Impedanz (*erster* *Pfeil*), die dann vor Erreichen des unteren Ösophagussphinkters wieder nach retrograd wandert (*zweiter* *Pfeil*). *Esophagus* Ösophagus, *Stomach* Magen

In der hochauflösenden Ösophagusmanometrie zeigt sich während des „gastric belching“, welches durch Aufstoßen von Luft aus dem Magen entsteht, kurz vor dem Eruktieren eine transiente Relaxation des unteren Ösophagussphinkters. Auf diese folgt die Relaxation des oberen Ösophagussphinkters. Da die Luft beim „supragastric belching“ erst von pharyngeal in den Ösophagus gepresst oder gesogen wird, zeigt sich hier vor der „Belching-Episode“ eine Relaxation des oberen Ösophagussphinkters anstelle des unteren Ösophagussphinkters. Der untere Ösophagussphinkter öffnet sich beim „supragastric belching“ nicht (Abb. 1 und 2; [28]).

**Abb. 2:**
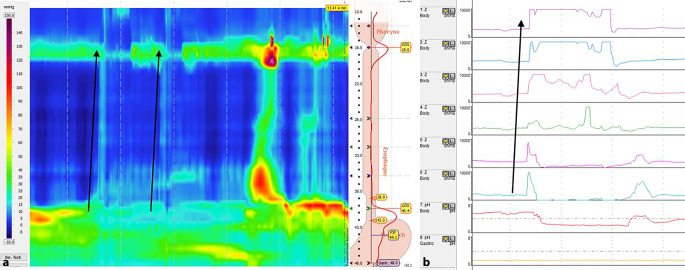
Gastrisches „belching“: **a** Hochauflösende Manometrie mit „clouse plot“. Relaxation des unteren Ösophagussphinkters vor der Relaxation des oberen Ösophagussphinkters. Ausbreitung der Luft in Richtung der *Pfeile*. **b** 24-h-Impedanz-pH-Metrie mit einem retrograden Anstieg der Impedanz vom Magen in den Ösophagus zum Pharynx. Miteinhergehend leichter Reflux (pH-Abfall im distalen Ösophagus). *Esophagus* Ösophagus, *Stomach* Magen

Bei der 24-h-Impedanz-pH-Metrie fällt das „gastric belching“ durch einen raschen retrograden Anstieg der Impedanz auf. Beim „supragastric belching“ zeigt sich im Gegensatz dazu erst ein anterograder Anstieg der Impedanz, welcher sich beim anschließenden Eruktieren der Luft vom distalen Ösophagus Richtung Pharynx wieder zur Baseline-Impedanz normalisiert (Abb. 1 und 2; [4, 30]).

„Supragastric belching“ ist auch in den 24-h-Impedanz-pH-Metrien gesunder Probanden nachweisbar und grundsätzlich nicht als pathologisch anzusehen. Bei asymptomatischen Patienten könnten bis zu 13 „Supragastric-Belching-Episoden“ pro 24 h noch als physiologisch eingeordnet werden [34]. Hierbei anzufügen ist jedoch auch, dass es 14 % der Patienten mit übermäßigem „belching“, definiert als >13 „Supragastric-Belching-Episoden“ pro 24 h, nicht bewusst war, unter einer erhöhten Anzahl an „Belching-Episoden“ zu leiden [34]. Dementsprechend ist – wie in den ROME-IV-Kriterien beschrieben – der klinische Kontext für die Diagnosestellung einer „belching disorder“ essenziell.

Neben einer Differenzierung des „supragastric“ und „gastric belching“ kann durch die 24-h-Impedanz-pH-Metrie auch die zeitliche Verteilung der „Belching-Episoden“ im Tagesverlauf und deren Zusammenhang mit anderen gastroösophagealen Symptomen wie Sodbrennen oder saurem Reflux beurteilt werden.

In der Pädiatrie hat sich die Impedanz-pH-Metrie zwischenzeitlich nebst der Refluxdiagnostik auch zur Diagnostik von Aerophagie und „belching disorders“, insbesondere beim „supragastric belching“, etabliert [57]. Die Impedanz-pH-Metrie ist dabei ein sehr nützliches Diagnoseinstrument zur diagnostischen Ein‑/Abgrenzung einer Refluxkrankheit versus Aerophagie, dem „belching“ und auch der Rumination. [57, 71].

### Therapie

Der erste Schritt in der Therapie des „supragastric belching“ besteht vor allem in der Aufklärung der Patienten über die zugrunde liegende Problematik. Dabei können oftmals auch die manometrischen Befunde bzw. die Ergebnisse der Impedanz-pH-Metrie nützlich sein, um die zugrunde liegende funktionelle Verhaltensstörung und die im weiteren Verlauf sinnvolle Verhaltenstherapien objektiv darlegen zu können [30].

Als effiziente Maßnahme hat sich in den letzten Jahren insbesondere die Verhaltenstherapie mit entsprechenden Atemübungen etabliert [3, 19, 30]. Im Vordergrund stehend ist hier die Verbesserung der Kontrolle des Diaphragmas, das speziell im Rahmen des zuvor beschriebenen „Air-Suction-Mechanismus“ für die Pathogenese des „supragastric belching“ von Bedeutung ist [30]. Auch bei Patienten mit PPI-resistenter GERD und gleichzeitig vorliegendem „supragastric belching“ konnte ein gutes Ansprechen einer standardisierten logopädischen Atemtherapie mit Fokus auf das Diaphragma demonstriert werden, einerseits in Hinblick auf die „Belching-Symptomatik“ selbst, andererseits auch auf die Refluxbeschwerden mit Verbesserung der Lebensqualität [47]. Das gleichzeitige Ansprechen auf die Refluxsymptomatik ist ein weiterer Indikator für den beschriebenen möglichen Zusammenhang der Refluxereignisse mit den meist kurzzeitig vorangehenden supragastrischen „Belching-Episoden“. Somit sollte bei einer Refluxerkrankung, insbesondere auch bei fehlendem Ansprechen auf eine PPI-Therapie, immer auch eine „Belching-Problematik“ evaluiert und gegebenenfalls therapiert werden [34, 47].

Von logopädischer Seite wird versucht, mögliche Auslöser des angewöhnten Habits einer übermäßigen „air suction“ oder „air injection“ zu identifizieren und diese abzubauen [43, 52] Therapeutische logopädische Maßnahmen sind hier unter anderem [43]:

- Erlernen einer korrekten abdominal-diaphragmalen Atmung
- Abbau von Hypertonus im Bereich des Mundbodens und Rachenraums, z. B. mittels Gähnübungen, entspannter Mund- oder Nasenatmung oder Übungen zur Erweiterung des Vokaltrakts
- Strategien für den Alltag bei akutem Auftreten von „Belching-Episoden“, wie zum Beispiel: Gähnen, entspannte Mundatmung, Umschalten auf abdominal-diaphragmale Atmung, Mundatmung mit „Atembremse“ (z. B. mit eingeklemmtem Bleistift zwischen den Zähnen oder durch Platzieren der Zungenspitze am Alveolarrand) [43, 66]

Die Wahl der Methoden und Techniken, die hierbei angewendet werden, richtet sich individuell nach den Patienten. Auch die Dauer und Frequenz der Therapie wird auf die Bedürfnisse des Patienten abgestimmt. In der Regel können erfolgreiche Kurzinterventionen im Sinne von zwei bis drei 60-minütigen Therapieeinheiten im Abstand von einem Monat durchgeführt werden, so wie dies beispielsweise am Universitätsspital Zürich praktiziert wird.

Sollte die logopädische Therapie keinen oder nur wenig Erfolg zeigen, kann eine zusätzliche physiotherapeutische Atemtherapie evaluiert werden [43].

Als medikamentöse Maßnahme eines „supragastric belching“ ist in der Literatur zudem die Gabe von Baclofen, einem γ‑Aminobuttersäure-Typ-B-Rezeptoragonisten, beschrieben. Es wird vermutet, dass dieser einerseits mit einer leichten Erhöhung des Tonus des unteren Ösophagussphinkters zu einer verminderten Anzahl von TLESR führt. Auf der anderen Seite ist eine Verminderung der diaphragmatischen Kontraktionen beschrieben, sodass man dies mit einer Verbesserung des „belching“ in Verbindung gesetzt hat. Aktuell ist diesbezüglich jedoch die Datenlage unzureichend, und auch in Hinblick auf das Nebenwirkungsspektrum kann daher keine Empfehlung zu dieser Pharmakotherapie beim „belching“ abgegeben werden [24].

## Singultus (Schluckauf, „hiccups“)

### Definition, Pathogenese und Ätiologie

Ein vom „belching“ abgrenzbares Krankheitsbild, welches in der Regel physiologisch intermittierend und zeitlich begrenzt auftritt, ist der Singultus. Der Terminus Singultus lässt sich aus dem Lateinischen ableiten und als Schluchzen oder Röcheln übersetzen. Beim Singultus kommt es zu einer reflektorischen Einatembewegung bei gleichzeitigem Stimmlippenverschluss. Der abrupte Stimmlippenverschluss während der Inspiration führt zum klassischen „Hicks-Geräusch“ [38]. Eine Einteilung des Singultus erfolgt nach seiner Dauer, wobei man einen Singultusanfall (< 48 h) von einem chronischen Singultus (≥ 48 h) unterscheidet [38]. Die pathophysiologische Bedeutung des Singultus ist bis zum jetzigen Zeitpunkt jedoch nicht vollständig verstanden:

Es wird jedoch angenommen, dass es sich hier um eine Störung des Schluckreflexbogens handelt. Daran beteiligt sind der N. phrenicus, der N. vagus, der Hirnstamm und der Sympathikus. Der Reflexbogen wird in 3 Teile gegliedert [10, 33, 38, 39, 48]:

1. Der afferente Schenkel setzt sich aus Fasern des N. vagus und N. phrenicus sowie thorakalen Anteilen des Sympathikus (Th6–Th12) zusammen.
2. Unter Beteiligung der verschiedenen Hirnnervenkerne sind Hirnstamm und Hypothalamus miteinander verschaltet. Ein eigentliches Schluckaufzentrum befindet sich in der Formatio reticularis der Medulla oblongata. So konnte in Tierversuchen durch eine elektrische Stimulation eines begrenzten Gebiets innerhalb der Formatio reticularis ein Schluckauf bei Katzen induziert werden.
3. Der efferente Schenkel steuert über den N. phrenicus Zwerchfellkontraktion, Kontraktionen der vorderen Skalenusmuskulatur (C5–7) sowie der Interkostalmuskulatur (Th1–12). Gleichzeitig wird ein Glottisschluss durch den N. vagus getriggert.

Ursächlich für einen anhaltenden Schluckauf kann eine Pathologie sein, welche einen oder mehrere Teilbereiche des Reflexbogens reizt und damit die Achse auslöst [10, 38, 39]. Hält ein Singultus längere Zeit an, kann dies zu einer starken Einschränkung der Lebensqualität führen. Hinter einem langanhaltenden Singultus können sich zudem ernsthafte Erkrankungen unterschiedlicher Organsysteme verbergen, die einer differenzierten sowie interdisziplinären diagnostischen Abklärung bedürfen. Denn Singultus ist ein Symptom, das durch eine Vielzahl von Ursachen ausgelöst werden kann, wovon einige ihren Ursprung im HNO-Bereich haben bzw. sich im HNO-Bereich manifestieren können (Tab. 1; [38]). Ein berühmter Patient mit persistierendem Schluckauf war beispielsweise Papst Pius XII., wobei die genaue Ursache für seinen Schluckauf ungeklärt bleibt [17]. Ein weiteres Beispiel ist der Fall eines 19-jährigen Patienten, welcher initial ausschließlich an einem hartnäckigen Singultus litt. Im Verlauf traten rechtsseitige Nackenbeschwerden nach Anstrengung, rechtsseitige Dysästhesien entlang der C7-Wurzel sowie Dysphagie und Dysphonie auf. Bei rechtsseitiger Parese der Stimmlippe konnte eine MRT-Untersuchung eine Typ-I-Arnold-Chiari-Malformation verbunden mit einer zervikothorakalen Syringomyelie nachgewiesen werden. Mittels eines ventrikuloperitonealen Shunts normalisierte sich nicht nur der neurologische Status, sondern es kam auch zu einem Sistieren des Singultus [39].

**Tab. 1:** Ursachen für chronischen Singultus [37]

**HNO-Ursachen**	**Abdominale Ursachen**	**Thorakale Ursachen**
Pharyngitis Laryngitis Struma Halstumoren zervikale Lymphadenopathie Fremdkörper im äußeren Gehörgang	*Magen* Magenkarzinom Gastritis Magendehnung Fremdkörper gastrointestinale Blutung *Pankreas* Pankreaskarzinom Pankreatitis *Leber und Gallenwege* Hepato- oder Splenomegalie Hepatitis Perihepatitis Cholezystitis Cholelithiasis Zirrhose *Darm* M. Crohn Colitis ulcerosa Darmobstruktion *Peritoneum* subphrenischer Abszess intraabdominaler Abszess Appendizitis Parasitose Peritonitis postoperativ *vaskulär* Aortenaneurysma *Harnwege* Hydronephrose Prostatainfektion oder -karzinom postoperativ	*Lunge und Bronchien* Pneumonie Bronchitis Tuberkulose Bronchialkarzinom Asthma *Pleura* Pleuritis Empyem *Mediastinum* Mediastinitis Tumoren Perikarditis Abszess *kardiovaskulär* Myokardinfarkt Angina pectoris thorakales Aortenaneurysma Cor pulmonale *Ösophagus* gastroösophagealer Reflux Ösophaguskarzinom oder -obstruktion Ösophagitis ösophageales Ulkus Hiatushernie *Zwerchfell* Zwerchfellhernie Zwerchfelltumoren Neurofibrom postoperativ
**Zentrales Nervensystem**	**Metabolische, infektiöse und toxische Ursachen**	**Psychiatrische Erkrankungen**
*Zerebrovaskuläre Erkrankungen* Hirninfarkt intrakranielle Blutung AV-Malformationen Arteriitis temporalis *entzündliche Erkrankungen* Meningitis Enzephalitis Neurosyphilis Hirnabszess Tuberkulome multiple Sklerose *Rückenmark* Syringomyelie Tabes dorsalis *Verschiedenes* Epilepsie Hydrozephalus VP-Shunt ZNS-Sarkoidose Schädel-Hirn-Trauma Neoplasien	Nierenversagen Diabetes mellitus Hyponatriämie Hypokalzämie Hypokapnie Hyperurikämie Insulin-Schocktherapie Fieber, septischer Schock Malaria Herpes zoster Typhus rheumatisches Fieber Influenza Alkohol *Medikamente* a‑Methyldopa Kortikosteroide Sulfonamide Benzodiazepine Barbiturate Ethosuximid Heroin Nikotin Etoposid	Trauerreaktion Hysterie Persönlichkeitsstörung Anorexia nervosa

Eine Zusammenfassung der wichtigsten Ursachen für einen Singultus findet sich in Tab. 1 aufgelistet, wobei in zwei Drittel der Fälle Pathologien des Gastrointestinaltrakts ursächlich sind, welche zumeist klinisch stumm verlaufen. Am häufigsten ist eine GERD wie auch eine akute Überblähung des Magens (z. B. durch große Mahlzeiten, kohlensäurehaltige Getränke oder Aerophagie etc.) [39]. Weitere häufige Ursachen für einen chronischen Singultus können einerseits Krankheiten des Magens, wie zum Beispiel eine Gastritis, ein Magenulkus oder ein Magenkarzinom sein. Andererseits können auch Pathologien des Ösophagus wie eine Ösophagitis, ein Ösophaguskarzinom oder eine Dehnung des Ösophagus ursächlich sein. Auch konnte ein Schluckauf bei gesunden Individuen provoziert werden, indem der proximale Ösophagus mit einem Ballon dilatiert wurde [11, 32, 51].

Tritt ein Singultus nach einem chirurgischen Eingriff auf, gilt es Komplikationen wie eine Peritonitis oder einen subphrenischen Abszess auszuschließen, die zu einem Reiz des N. phrenicus führen. Ein postoperativer Singultus tritt in der Regel innerhalb der ersten vier Tage nach Operation auf [11, 32, 51]. In Zusammenschau der möglichen Ätiologie ist ergänzend zu erwähnen, dass bei fortgeschrittenen Malignomen ein (i. d. R. multifaktorieller) Singultus häufig und therapieresistent ist [11, 32, 51]. Auf die zahlreichen weiteren Ursachen kann aufgrund des großen Umfangs derselben an dieser Stelle nicht weiter eingegangen werden.

### Diagnostik

Der akute Singultus beim Gesunden ist meistens selbstlimitierend und bedarf keiner weiteren Diagnostik. Eine Ausnahme bildet eine atypische Manifestation eines Herzinfarkts, welcher bei anhaltendem Singultus immer ausgeschlossen werden sollte. Bei einem chronischen Singultus ist die Indikation zur weiteren Abklärung immer gegeben, da schwere Krankheiten ursächlich sein können.

Die Diagnostik des Singultus beginnt primär mit einer ausführlichen Anamnese, die folgende Punkte beinhaltet [38, 74]:

- Wie lange besteht der Singultus und ist die Dauer >48h?
- Besteht eine Persistenz während des Schlafs (ein psychogener Singultus sistiert immer im Schlaf)?
- Zeigen sich Auswirkungen auf den Alltag (Beeinträchtigung der Nahrungs‑/Flüssigkeitsaufnahme, Ausmaß der psychischen Belastung)?
- Liegen Begleitsymptome vor (Systemanamnese: Gastrointestinaltrakt, Respirationstrakt etc.)?
- Lässt sich der Singultus durch bestimmte Manöver beeinflussen?
- Gibt es einen zeitlichen Zusammenhang mit einer Operation (postoperativer Singultus)?
- Wurde neue Medikamente eingenommen?

Im Anschluss an die Anamnese sollten im HNO-ärztlichen Setting ein kompletter HNO- und Neurostatus erfasst, eine Halssonographie sowie eine Laboruntersuchung durchgeführt werden. Essenziell ist bei der HNO-ärztlichen Untersuchung die Inspektion der äußeren Gehörgänge, da ein Fremdkörper durch eine Reizung des R. auricularis des N. vagus respektive des N. auricularis magnus des sensiblen Asts einen Singultus induzieren kann. Mittels Halssonographie sollten strukturelle Ursachen im Verlauf des N. vagus und N. phrenicus ausgeschlossen werden. Eine primäre Labordiagnostik beinhaltet ein Standardblutbild, den CRP-Wert, Elektrolyte, Harnstoff, Kreatinin, Leberwerte und bei auffälliger abdominaler Anamnese die Amylase oder Lipase [35, 38, 74].

Nach erfolgter HNO-ärztlicher Untersuchung sollte der Patient zur Komplettierung des internistischen Status (Thorax- und Abdomenstatus) und Ausschluss eines atypischen Herzinfarkts an einen Internisten zugewiesen werden. Die weitere Diagnostik richtet sich nach der vermuteten Ätiologie in Zusammenschau der erhobenen Befunde [13, 20, 35, 45].

### Therapie

Grundsätzlich entspricht die Therapie des Singultus der Behandlung der zugrunde liegenden Erkrankung. Wenn sich in der Diagnostik keine Ursache identifizieren lassen sollte, so können primär physische Manöver, sekundär medikamentöse Therapien oder tertiär operative Maßnahmen evaluiert werden.

Bei den physischen Manövern wird primär auf eine Unterbrechung der Reflexachse durch Induzieren einer Hyperkapnie beziehungsweise vagalen oder diaphragmalen Reizung abgezielt.

Mögliche Manöver sind [1, 50]:

- Luft anhalten/CO_2_-Rückatmung
- Stimulation Rachenhinterwand/Massage harter/weicher Gaumen
- Zunge herausziehen
- Kompression: Bulbus, N. phrenicus, Karotis
- Anhaltendes Valsalva-Manöver
- Flüssigkeit durch kleinlumige Strohalme trinken
- Trinken eines Glases Eiswasser in gebückter Körperhaltung mit Kopf nach vorn gebeugt
- Erschrecken
- Kalte Ohrspülungen
- Rektale Stimulation

Sollten diese therapeutischen Maßnahmen zu keinem Ansprechen führen, so könnte eine medikamentöse Behandlungsstrategie diskutiert werden. Das antidopaminerg wirksame Neuroleptikum Chlorpromazin ist das einzige durch die US Food and Drug Administration (FDA) für die Behandlung von chronischem Singultus zugelassene Medikament, aufgrund der zahlreichen Nebenwirkungen jedoch nicht als Mittel erster Wahl empfohlen. [64, 75]. Primär wird eine Kombinationstherapie aus Baclofen, einem PPI sowie einem Prokinetikum empfohlen [55, 75]. Baclofen dämpft sowohl die mono- als auch die polysynaptische Reflexübertragung im Rückenmark durch Stimulation der GABA_B_-Rezeptoren [55, 75]. Unterstützt werden kann diese Therapie mit einem Protonenpumpeninhibitor (PPI), wodurch der gastroösophageale Reflux als häufigste Ursache des Singultus behandelt wird [12]. Bei unzureichendem Therapieansprechen kann Carbamazepin ergänzt werden. Carbamazepin hat einen synergistischen Effekt zum Baclofen durch Hemmung exzitatorischer Potenziale im ZNS. Wichtig ist, dass Baclofen nach Beendigung der Therapie ausgeschlichen werden muss und dass die Carbamazepingabe mit regelmäßigen Laborkontrollen (Blutbild, Leberparameter, Natriumwerte) kombiniert werden sollte [64].

Bei positivem Ansprechen eines Singultus auf eine medikamentöse Therapie soll diese nach 2 Wochen gestoppt werden. Bei Wiederauftreten des Singultus ist eine kontinuierliche medikamentöse Therapie zu erwägen [64, 75].

Ultima Ratio bei frustraner nichtmedikamentöser und medikamentöser Therapie sind chirurgische Eingriffe wie beispielsweise eine Phrenikotomie oder eine Vagusnervstimulation, welche den Reflexbogen des Schluckaufs unterbinden sollen. Hierbei sind jedoch präoperative Abklärungen in Hinblick auf die exakte Lokalisation der Reflexbogenreizung sowie respiratorischen Reserve angezeigt [49, 70].

Akuter Schluckauf bei Kindern ist in der Regel gutartig und selbstlimitierend. Anhaltender oder hartnäckiger Schluckauf kann jedoch auch bei der pädiatrischen Population ein Anzeichen für eine ernsthafte Erkrankung sein. Die zugrunde liegende Ursache sollte nach Möglichkeit gesucht und behandelt werden. Es gibt allerdings keine offiziellen Leitlinien für die Behandlung von Singultus bei Kindern. [72].

## Aerophagie

### Definition, Pathogenese und Ätiologie

Abzugrenzen ist das „belching“ vom Begriff der Aerophagie, welche das Schlucken von exzessiven Mengen an Luft beschreibt und die pädiatrische, aber auch die Erwachsenenpopulation betreffen kann [15]. Bei Kindern ist kürzlich je nach Region und je nach zugrunde liegender Definition eine Prävalenz der Aerophagie von 0,4 bis 18 % beschrieben worden [54]. Eine ausgeprägte Aerophagie mit Schlucken großer Mengen Luft und abdominalen Komplikationen beispielsweise im Sinne eines Ileus ist jedoch sehr selten und vor allem bei syndromalen Patienten beschrieben worden [18, 68].

Das Resultat der Aerophagie ist eine vermehrte Ansammlung von Gas im gastroduodenalen Raum, mit typischerweise Zunahme über den Tagesverlauf. Eine abdominale Röntgenuntersuchung mit Nachweis eines Normalbefundes morgens und luftgefülltem Gastrointestinaltrakt gegen Abend hin ist pathognomonisch. Die Hauptsymptome sind abdominales Völlegefühl und Blähungen sowie gastrisches „belching“ [30]. Entgegen der früher oftmals vertretenen Meinung ist die Aerophagie keine Ursache des „supragastric belching“, kann aber damit assoziiert sein. [52]. Eine Aerophagie kann auch mit einer R‑CPD verwechselt werden. Es lohnt sich daher immer, den Patienten danach zu fragen, ob er oder sie Luft aufstoßen kann.

### Diagnostik

**Abb. 3:**
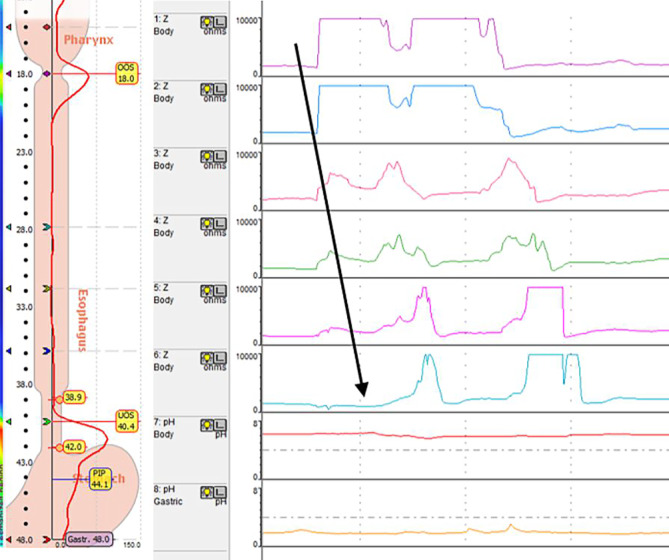
Aerophagie diagnostiziert mittels 24-h-Impedanz-pH-Metrie mit anterogradem Anstieg der Impedanz vom Pharynx bis in den Magen entlang des *Pfeiles* als Nachweis eines Schluckens von Luft. *Esophagus* Ösophagus, *Stomach* Magen

Für die Diagnostik der Aerophagie steht ebenfalls die 24-h-Impedanz-pH-Metrie zur Verfügung [7]. Während die Luft durch die ösophageale Peristaltik Richtung Magen befördert wird, ist ein anterograder Anstieg der Impedanz zu beobachten. Der zeitliche Anstieg der Impedanz entlang der Sonde verläuft im Vergleich zum „supragastric belching“ bei der Aerophagie von Luft deutlich langsamer und nimmt mehrere Sekunden in Anspruch (Abb. 3; [5]). Bei vermehrt vorkommenden Aerophagieepisoden werden häufig konsekutiv auftretende gastrische „Belching-Phasen“ in der 24-h-Impedanz-pH-Metrie beobachtet. Dieses diagnostische Verfahren wird ebenso in der Pädiatrie angewendet, in Situationen, in denen die Klinik unklar ist, respektive zur besseren Abgrenzung gegenüber anderen ösophagealen Funktionsstörungen [21].

### Therapie

Eine Therapie der Aerophagie basiert weitgehend auf individuellen Expertenmeinungen und verfolgt wie beim „belching“ verhaltenstherapeutische Ansätze. Eine evidenzbasierte Empfehlung kann bei fehlender Daten- und Studienlage nicht ausgesprochen werden. Von logopädischer Seite könnte eine genaue Aufklärung des Patienten über den zugrunde liegenden Pathomechanismus des Luftschluckens und das nicht optimale Schluckmuster erfolgen. Ebenfalls kann das Erlernen eines korrekten Schluckmusters und dessen Transfer in den Alltag angestrebt werden. Daneben könnten Maßnahmen wie die Aufnahme von lediglich kleineren Portionen Nahrung oder die Reduzierung des Esstempos hilfreich sein [3].

## Ruminationssyndrom

Das nach ROME IV definierte Ruminationssyndrom ist durch rezidivierende postprandiale Regurgitationen von Nahrung und Flüssigkeit charakterisiert [14, 41]. Dieses Zustandsbild kann bei allen Altersgruppen von pädiatrischen bis erwachsenen Patienten auftreten. Die Prävalenz des Ruminationssyndroms liegt gemäß ROME Foundation bei 2,8 % in der Allgemeinbevölkerung [63]. Eine pädiatrische Studie aus Sri Lanka hat bei 10- bis 16-jährigen Jugendlichen eine höhere Prävalenz mit bis zu 5 % beschrieben [53].

**Abb. 4:**
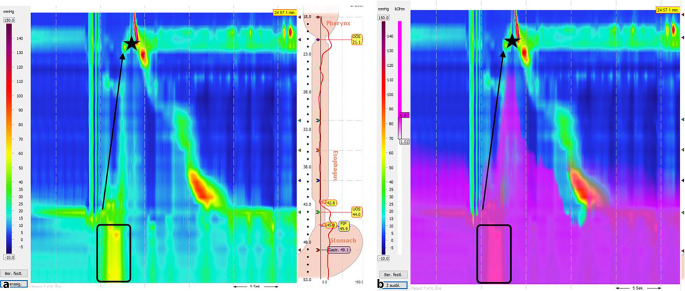
**a**, **b** Hochauflösende Manometrie mit „clouse plot“ bei Ruminationssyndrom: Abdominaler Druckanstieg (*schwarzes* *Kästchen*) mit anschließender retrograder Flussrichtung des Mageninhalts (*Pfeil*). *Stern *Regurgitation des Mageninhalt. **b** entspricht dabei einem modifizierten „clouse plot“ mit Impedanz. *Violette Anfärbung* entspricht Flüssigkeit mit hoher Leitfähigkeit. *Esophagus* Ösophagus, *Stomach* Magen

Die Diagnose des Ruminationssyndroms wird primär klinisch gestellt. Es handelt sich um ein Tic-ähnliches Zustandsbild, mit willkürlichem, aber unbewusst ablaufendem, wiederkehrendem Regurgitieren von Mageninhalt. Idealerweise wird im Rahmen einer Diagnostik eine ösophageale Manometrie mit Ruminationsprotokoll durchgeführt, bei dem der Patient eine die typischen Symptome auslösende Mahlzeit einnimmt und für 30 min nachbeobachtet wird (Abb. 4). Meist beginnen die Regurgitationsepisoden beim Ruminationssyndrom während oder kurz nach den Mahlzeiten und halten für bis zu 2 h an. Im Gegensatz zum gastroösophagealen Reflux wird das regurgitierte Material oft als nicht sauer oder bitter beschrieben und ist meist noch deutlich als die zuvor eingenommene Nahrung zu identifizieren [46]. Auch in der Pädiatrie nimmt der Stellenwert der hochauflösenden Ösophagusmanometrie in der Diagnostik des Ruminationssyndroms zu. Die diagnostischen Merkmale sind die gleichen wie bei den Erwachsenen [56, 58, 61].

In der 24-h-Impedanz-pH-Metrie zeigt sich beim Ruminationssyndrom ein Abfall der Impedanz in retrograder Richtung. Dieses ähnelt dem Impedanzmuster bei einer gastroösophagealen Refluxerkrankung, sodass eine sichere Differenzierung mit dieser Untersuchungsmethode nicht sicher gewährleistet ist [29]. Eine neuere Studie beschreibt allerdings einen MII-pH-basierten Ruminations-Score (postprandiale nichtsaure Refluxepisoden/Stunde und assoziierte Symptome), wo in 46 % der Patienten mit therapierefraktärer GERD mit einer Sensitivität von 91,7 % und Spezifität von 78,6 % die Diagnose des Ruminationssyndroms gestellt werden konnte [44].

Ruminationsepisoden können entweder primär auftreten oder sekundär durch gastroösophageale Refluxepisoden oder „Supragastric-Belching-Episoden“ getriggert werden [60]. Differenzialdiagnostisch ist bei rezidivierenden postprandialen Regurgitationen auch an eine Achalasie, eine ösophagogastrale „outflow obstruction“ (EGJOO), eine Gastroparese oder an Essstörungen, wie beispielsweise eine Bulimia nervosa zu denken. Zu deren Differenzierung müssen weitere anamnestische Informationen und diagnostische Befunde herangezogen werden. Ein Ansprechen auf eine antisekretorische Therapie mit einem PPI spricht eher für eine gastroösophageale Refluxerkrankung. Bei einer Achalasie oder einer EGJOO werden hingegen dysphagische Symptome beklagt, und bei einer Gastroparese zeigen sich vor den Regurgitationsepisoden häufig Nausea und ein ausgeprägtes Völlegefühl. Eine Abgrenzung zu einer Essstörung kann sich ebenfalls schwierig gestalten, da es auch beim Ruminationssyndrom zu Gewichtsverlust kommen kann [40, 46]. Zudem können diese Krankheitsbilder ebenfalls mit anderen Erkrankungen überlappen, die eine Abklärung durch Spezialisten anderer Fachdisziplinen erfordern [16, 36].

## Fazit für die Praxis

- Bei einer „Belching-Erkrankung“ ist im Gegensatz zum „gastric belching“ vor allem das „supragastric belching“ mit Beschwerden und einem Leidensdruck verbunden.
- „Supragastric belching“ und Ruminationen werden häufig im Rahmen einer Refluxerkrankung fehlinterpretiert und zeigen oftmals kein suffizientes Ansprechen auf eine PPI-Therapie.
- Das „Inability-to-belch-Syndrom“ (R-CPD) ist eine wichtige Differenzialdiagnose bei Aerophagie mit therapeutisch signifikanten Konsequenzen für den HNO-Arzt.
- Eine hochauflösende Ösophagusmanometrie (HRM) und 24-h-Impedanz-pH-Metrie sind wichtige Werkzeuge zur Diagnosesicherung bzw. zur Differenzierung einer Refluxerkrankung, eines „belching“, einer Aerophagie oder eines Ruminationssyndroms.
- Therapeutisch kann beim „belching“ oder Ruminationssyndrom eine Verhaltenstherapie beispielsweise mit diaphragmatischen Atemübungen durchgeführt werden, welche bei vielen Betroffenen eine deutliche Symptomverbesserung erzielen kann.
- Sollte eine PPI-resistente Refluxerkrankung vorliegen und gleichzeitig eine „Belching-Symptomatik“ beklagt werden, so kann eine „Belching-Therapie“ auch zur Verbesserung der Refluxsymptomatik evaluiert werden.
- Bei einem persistierenden Singultus sollte immer auch eine schwerwiegende Grunderkrankung als Auslöser ausgeschlossen werden.

## References

[1] Alvarez J, Anderson JM, Snyder PL et al (2021) Evaluation of the forced inspiratory suction and swallow tool to stop hiccups. Jama Netw Open 4:e2113933 34143196 10.1001/jamanetworkopen.2021.13933 PMC8214157

[2] Bastian RW, Smithson ML (2019) Inability to belch and associated symptoms due to retrograde cricopharyngeus dysfunction: diagnosis and treatment. OTO Open 3:2473974X19834553 31236539 10.1177/2473974X19834553 PMC6572913

[3] Bredenoord AJ (2013) Management of belching, hiccups, and aerophagia. Clin Gastroenterol Hepatol 11:6–12 22982101 10.1016/j.cgh.2012.09.006

[4] Bredenoord AJ, Weusten BL, Sifrim D et al (2004) Aerophagia, gastric, and supragastric belching: a study using intraluminal electrical impedance monitoring. Gut 53:1561–1565 15479671 10.1136/gut.2004.042945 PMC1774303

[5] Bredenoord AJ, Weusten BL, Timmer R et al (2005) Relationships between air swallowing, intragastric air, belching and gastro-oesophageal reflux. Neurogastroenterol Motil 17:341–347 15916621 10.1111/j.1365-2982.2004.00626.x

[6] Bredenoord AJ, Weusten BL, Timmer R et al (2006) Air swallowing, belching, and reflux in patients with gastroesophageal reflux disease. Am J Gastroenterol 101:1721–1726 16817838 10.1111/j.1572-0241.2006.00687.x

[7] Bredenoord AJ, Weusten BL, Timmer R et al (2005) Reproducibility of multichannel intraluminal electrical impedance monitoring of gastroesophageal reflux. Am J Gastroenterol 100:265–269 15667480 10.1111/j.1572-0241.2005.41084.x

[8] Chial HJ, Camilleri M, Williams DE et al (2003) Rumination syndrome in children and adolescents: diagnosis, treatment, and prognosis. Pediatrics 111:158–162 12509570 10.1542/peds.111.1.158

[9] Conchillo JM, Selimah M, Bredenoord AJ et al (2007) Air swallowing, belching, acid and non-acid reflux in patients with functional dyspepsia. Aliment Pharmacol Ther 25:965–971 17403001 10.1111/j.1365-2036.2007.03279.x

[10] Davis JN (1970) An experimental study of hiccup. Brain 93:851–872 5490279 10.1093/brain/93.4.851

[11] De Hoyos A, Esparza EA, Cervantes-Sodi M (2010) Non-erosive reflux disease manifested exclusively by protracted hiccups. J Neurogastroenterol Motil 16:424–427 21103425 10.5056/jnm.2010.16.4.424 PMC2978396

[12] Dore MP, Pedroni A, Pes GM et al (2007) Effect of antisecretory therapy on atypical symptoms in gastroesophageal reflux disease. Dig Dis Sci 52:463–468 17211695 10.1007/s10620-006-9573-7

[13] Doshi H, Vaidyalingam R, Buchan K (2008) Atrial pacing wires: an uncommon cause of postoperative hiccups. Br J Hosp Med 69:534 10.12968/hmed.2008.69.9.31053 18819310

[14] Drossman DA (2016) Functional gastrointestinal disorders: history, pathophysiology, clinical features and rome IV. Gastroenterology 10.1053/j.gastro.2016.02.032 27144617

[15] Drossman DA (2016) Functional gastrointestinal disorders: what’s new for Rome IV? Lancet Gastroenterol Hepatol 1:6–8 28404114 10.1016/S2468-1253(16)30022-X

[16] Eckern M, Stevens W, Mitchell J (1999) The relationship between rumination and eating disorders. Int J Eat Disord 26:414–419 10550782 10.1002/(SICI)1098-108X(199912)26:4<414::AID-EAT7>3.0.CO;2-8

[17] Fodstad H, Nilsson S (1993) Intractable singultus: a diagnostic and therapeutic challenge. Br J Neurosurg 7:255–260 8338646 10.3109/02688699309023807

[18] Frye RE, Hait EJ (2006) Air swallowing caused recurrent ileus in Tourette’s syndrome. Pediatrics 117:e1249–e1252 16651280 10.1542/peds.2005-2914 PMC2587247

[19] Glasinovic E, Wynter E, Arguero J et al (2018) Treatment of supragastric belching with cognitive behavioral therapy improves quality of life and reduces acid gastroesophageal reflux. Am J Gastroenterol 113:539–547 29460918 10.1038/ajg.2018.15

[20] Habadi MI, Hamza N, Abdalla Balla TH et al (2021) Persistent hiccups as presenting symptom of COVID-19: a case of 64-year-old male from international medical center, Jeddah, Saudi Arabia. Cureus 13:e20158 35003987 10.7759/cureus.20158 PMC8723778

[21] Halb C, Pomerleau M, Faure C (2014) Multichannel intraesophageal impedance pattern of children with aerophagia. Neurogastroenterol Motil 26:1010–1014 24796405 10.1111/nmo.12355

[22] Hemmink GJ, Bredenoord AJ, Weusten BL et al (2009) Supragastric belching in patients with reflux symptoms. Am J Gastroenterol 104:1992–1997 19455107 10.1038/ajg.2009.203

[23] Herbst J, Friedland GW, Zboralske FF (1971) Hiatal hernia and “rumination” in infants and children. J Pediatr 78:261–265 5539770 10.1016/S0022-3476(71)80009-4

[24] Jeong SO, Lee JS, Lee TH et al (2021) Characteristics of symptomatic belching in patients with belching disorder and patients who exhibit gastroesophageal reflux disease with belching. J Neurogastroenterol Motil 27:231–239 33424014 10.5056/jnm20114 PMC8026376

[25] Karagama Y (2021) Abelchia: inability to belch/burp—a new disorder? Retrograde cricopharyngeal dysfunction (RCPD). Eur Arch Otorhinolaryngol 278:5087–5091 33893849 10.1007/s00405-021-06790-w PMC8553696

[26] Karamanolis G, Triantafyllou K, Tsiamoulos Z et al (2010) Effect of sleep on excessive belching: a 24-hour impedance-pH study. J Clin Gastroenterol 44:332–334 19834335 10.1097/MCG.0b013e3181bd885e

[27] Keeratichananont S, Patcharatrakul T, Gonlachanvit S (2023) Gastroesophageal reflux characteristics in supragastric belching patients with positive versus negative pH monitoring: an evidence of secondary gastroesophageal reflux disease from excessive belching. J Neurogastroenterol Motil 29:343–351 37417261 10.5056/jnm22198 PMC10334197

[28] Kessing BF, Bredenoord AJ, Smout AJ (2012) Mechanisms of gastric and supragastric belching: a study using concurrent high-resolution manometry and impedance monitoring. Neurogastroenterol Motil 24:e573–579 23072402 10.1111/nmo.12024

[29] Kessing BF, Bredenoord AJ, Smout AJ (2014) Objective manometric criteria for the rumination syndrome. Am J Gastroenterol 109:52–59 24366235 10.1038/ajg.2013.428

[30] Kessing BF, Bredenoord AJ, Smout AJ (2014) The pathophysiology, diagnosis and treatment of excessive belching symptoms. Am J Gastroenterol 109:1196–1203 25001253 10.1038/ajg.2014.165

[31] Kessing BF, Bredenoord AJ, Velosa M et al (2012) Supragastric belches are the main determinants of troublesome belching symptoms in patients with gastro-oesophageal reflux disease. Aliment Pharmacol Ther 35:1073–1079 22428801 10.1111/j.1365-2036.2012.05070.x

[32] Khorakiwala T, Arain R, Mulsow J et al (2008) Hiccups: an unrecognized symptom of esophageal cancer? Am J Gastroenterol 103:801 18341501 10.1111/j.1572-0241.2007.01612_4.x

[33] Kolodzik PW, Eilers MA (1991) Hiccups (singultus): review and approach to management. Ann Emerg Med 20:565–573 2024799 10.1016/S0196-0644(05)81620-8

[34] Koukias N, Woodland P, Yazaki E et al (2015) Supragastric belching: prevalence and association with gastroesophageal reflux disease and esophageal hypomotility. J Neurogastroenterol Motil 21:398–403 26130635 10.5056/jnm15002 PMC4496903

[35] Krysiak W, Szabowski S, Stepien M et al (2008) Hiccups as a myocardial ischemia symptom. Pol Arch Med Wewn 118:148–151 18476462 10.20452/pamw.338

[36] Larocca FE, Della-Fera MA (1986) Rumination: its significance in adults with bulimia nervosa. Psychosomatics 27:209–212 3457391 10.1016/S0033-3182(86)72713-8

[37] Launois S, Bizec JL, Whitelaw WA et al (1993) Hiccup in adults: an overview. Eur Respir J 6:563–575 8491309 10.1183/09031936.93.06040563

[38] Lehnert H (2020) DGIM Innere Medizin. Springer, Berlin

[39] Loft LM, Ward RF (1992) Hiccups. A case presentation and etiologic review. Arch Otolaryngol Head Neck Surg 118:1115–1119 1389062 10.1001/archotol.1992.01880100107020

[40] Malcolm A, Thumshirn MB, Camilleri M et al (1997) Rumination syndrome. Mayo Clin Proc 72:646–652 9212767 10.1016/S0025-6196(11)63571-4

[41] Martinez M, Rathod S, Friesen HJ et al (2021) Rumination syndrome in children and adolescents: a mini review. Front Pediatr 9:709326 34490165 10.3389/fped.2021.709326 PMC8416921

[42] Masui D, Nikaki K, Sawada A et al (2022) Belching in children: prevalence and association with gastroesophageal reflux disease. Neurogastroenterol Motil 34:e14194 34190371 10.1111/nmo.14194

[43] Moshiree B, Drossman D, Shaukat A (2023) AGA clinical practice update on evaluation and management of belching, abdominal bloating, and distention: expert review. Gastroenterology 165:791–800 (e793) 37452811 10.1053/j.gastro.2023.04.039

[44] Nakagawa K, Sawada A, Hoshikawa Y et al (2019) Persistent postprandial regurgitation vs rumination in patients with refractory gastroesophageal reflux disease symptoms: identification of a distinct rumination pattern using ambulatory impedance-pH monitoring. Am J Gastroenterol 114:1248–1255 31246694 10.14309/ajg.0000000000000295

[45] Ng JL, Aziz EF, Herzog E (2011) Electrocardiogram for hiccups? Am J Med 124:e5–e6 21092925 10.1016/j.amjmed.2010.06.025

[46] O’brien MD, Bruce BK, Camilleri M (1995) The rumination syndrome: clinical features rather than manometric diagnosis. Gastroenterology 108:1024–1029 7698568 10.1016/0016-5085(95)90199-X

[47] Ong AM, Chua LT, Khor CJ et al (2018) Diaphragmatic breathing reduces belching and proton pump inhibitor refractory gastroesophageal reflux symptoms. Clin Gastroenterol Hepatol 16:407–416 (e402) 29104130 10.1016/j.cgh.2017.10.038

[48] Oshima T, Sakamoto M, Arita H (1994) Hiccuplike response elicited by mechanical stimulation of dorsal epipharynx of cats. J Appl Physiol 76:1888–1895 (1985) 8063646 10.1152/jappl.1994.76.5.1888

[49] Payne BR, Tiel RL, Payne MS et al (2005) Vagus nerve stimulation for chronic intractable hiccups. Case report. J Neurosurg 102:935–937 15926725 10.3171/jns.2005.102.5.0935

[50] Petroianu GA (2015) Treatment of hiccup by vagal maneuvers. J Hist Neurosci 24:123–136 25055206 10.1080/0964704X.2014.897133

[51] Pooran N, Lee D, Sideridis K (2006) Protracted hiccups due to severe erosive esophagitis: a case series. J Clin Gastroenterol 40:183–185 16633116 10.1097/00004836-200603000-00002

[52] Popa SL, Surdea-Blaga T, David L et al (2022) Supragastric belching: pathogenesis, diagnostic issues and treatment. Saudi J Gastroenterol 28:168–174 35562166 10.4103/sjg.sjg_405_21 PMC9212115

[53] Rajindrajith S, Devanarayana NM, Crispus Perera BJ (2012) Rumination syndrome in children and adolescents: a school survey assessing prevalence and symptomatology. BMC Gastroenterol 12:163 23157670 10.1186/1471-230X-12-163 PMC3538663

[54] Rajindrajith S, Gunawardane D, Kuruppu C et al (2022) Epidemiology of aerophagia in children and adolescents: a systematic review and meta-analysis. PLoS ONE 17:e271494 35905055 10.1371/journal.pone.0271494 PMC9337652

[55] Ramirez FC, Graham DY (1992) Treatment of intractable hiccup with baclofen: results of a double-blind randomized, controlled, cross-over study. Am J Gastroenterol 87:1789–1791 1449142

[56] Righini Grunder F, Aspirot A, Faure C (2017) High-resolution esophageal manometry patterns in children and adolescents with rumination syndrome. J Pediatr Gastroenterol Nutr 65:627–632 29072581 10.1097/MPG.0000000000001618

[57] Rosen R (2022) Novel advances in the evaluation and treatment of children with symptoms of gastroesophageal reflux disease. Front Pediatr 10:849105 35433543 10.3389/fped.2022.849105 PMC9010502

[58] Rosen R, Rodriguez L, Nurko S (2017) Pediatric rumination subtypes: a study using high-resolution esophageal manometry with impedance. Neurogastroenterol Motil 29:10.1111/nmo.12998 PMC5393952 28002887

[59] Runggaldier D, Colotto-Vith U, Pohl D et al (2023) Help, i can’t burp! a brief overview and case discussion of retrograde cricopharyngeal dysfunction. HNO 10.1007/s00106-023-01383-x PMC10827936 37861741

[60] Sasegbon A, Hasan SS, Disney BR et al (2022) Rumination syndrome: pathophysiology, diagnosis and practical management. Frontline Gastroenterol 13:440–446 36046491 10.1136/flgastro-2021-101856 PMC9380772

[61] Singendonk MMJ, Oors JM, Bredenoord AJ et al (2017) Objectively diagnosing rumination syndrome in children using esophageal pH-impedance and manometry. Neurogastroenterol Motil 29:10.1111/nmo.12996 28078818

[62] Soykan I, Chen J, Kendall BJ et al (1997) The rumination syndrome: clinical and manometric profile, therapy, and long-term outcome. Dig Dis Sci 42:1866–1872 9331149 10.1023/A:1018854925196

[63] Sperber AD, Bangdiwala SI, Drossman DA et al (2021) Worldwide prevalence and burden of functional gastrointestinal disorders, results of rome foundation global study. Gastroenterology 160:99–114 (e113) 32294476 10.1053/j.gastro.2020.04.014

[64] Steger M, Schneemann M, Fox M (2015) Systemic review: the pathogenesis and pharmacological treatment of hiccups. Aliment Pharmacol Ther 42:1037–1050 26307025 10.1111/apt.13374

[65] Tack J, Talley NJ, Camilleri M et al (2006) Functional gastroduodenal disorders. Gastroenterology 130:1466–1479 16678560 10.1053/j.gastro.2005.11.059

[66] Ten Cate L, Herregods TVK, Dejonckere PH et al (2018) Speech therapy as treatment for supragastric belching. Dysphagia 33:707–715 29574541 10.1007/s00455-018-9890-y

[67] Thumshirn M, Camilleri M, Hanson RB et al (1998) Gastric mechanosensory and lower esophageal sphincter function in rumination syndrome. Am J Physiol 275:G314–321 9688659 10.1152/ajpgi.1998.275.2.G314

[68] Van Der Kolk MB, Bender MH, Goris RJ (1999) Acute abdomen in mentally retarded patients: role of aerophagia. Report of nine cases. Eur J Surg 165:507–511 10391173 10.1080/110241599750006802

[69] Vandenplas Y, Salvatore S, Devreker T et al (2007) Gastro-oesophageal reflux disease: oesophageal impedance versus pH monitoring. Acta Paediatr 96:956–962 17498193 10.1111/j.1651-2227.2007.00306.x

[70] Weeks C (1931) Surgery of the phrenic nerve in treatment of intractable hiccup. Ann Surg 93:811–815 17866535 10.1097/00000658-193104000-00002 PMC1398408

[71] Woodley FW, Ciciora SL, Vaz K et al (2020) Novel use of impedance technology shows that esophageal air events can be temporally associated with gastroesophageal reflux disease-like symptoms. J Pediatr Gastroenterol Nutr 70:e7–e11 31880681 10.1097/MPG.0000000000002514

[72] Woodley FW, Williams K, Di Lorenzo C et al (2022) Significant temporal association of esophageal air events (supragastric belches, air swallows, and gastric belches) with hiccups: a case study in an adolescent. JPGN Rep 3:e209 37168628 10.1097/PG9.0000000000000209 PMC10158523

[73] Yadlapati R, Tye M, Roman S et al (2018) Postprandial high-resolution impedance manometry identifies mechanisms of nonresponse to proton pump inhibitors. Clin Gastroenterol Hepatol 16:211–218 (e211) 28911949 10.1016/j.cgh.2017.09.011 PMC5794564

[74] Zenk Pa F (1999) Singultus. HNO 47:867–875 10550370 10.1007/s001060050527

[75] Zhang C, Zhang R, Zhang S et al (2014) Baclofen for stroke patients with persistent hiccups: a randomized, double-blind, placebo-controlled trial. Trials 15:295 25052238 10.1186/1745-6215-15-295 PMC4223604

